# Supplementary material for: Prefabrication of a ribosomal protein subcomplex essential for eukaryotic ribosome formation
Source: eLife. 2016 Dec 8;5:e21755. doi: 10.7554/eLife.21755 (PMC5148605; doi:10.7554/eLife.21755)
Supplement: Supplementary file 1. — (A) Yeast strains used in this study. (B) Plasmids used in this study. DOI: http://dx.doi.org/10.7554/eLife.21755.012 [file elife-21755-supp1.docx]

**Supplementary File 1**

**Supplementary File 1A - Yeast strains used in this study**

| **Strain name** | **Genotype** | **Origin** |
| --- | --- | --- |
| BY4741 | *MATa ura3 his3 leu2 met15 TRP1* | Euroscarf |
| W303 | *MATa ade2 ura3 his3 leu2 trp1* | ([Pertschy et al., 2007](#_ENREF_6)) |
| P*_GAL1_-TSR2* | *MATa ura3 his3 leu2 met15 TRP1 Gal1-TSR2::natNT2* | ([Schütz et al., 2014](#_ENREF_8)) |
| P*_GAL1_-FAP7* | *MATa ura3 his3 leu2 met15 TRP1 Gal1-FAP7::natNT2* | This study |
| P*_GAL1_-rps14a∆ rps14b∆* | *MATα ura3 his3 leu2 met15 TRP1 rps14a::KANMX rps14b::LEU2 pRS316-Gal1-RPS14A::URA3* | Jakovljevic et al., 2004 |
| Enp1-TAP | *MATa ura3 leu2 TRP1 ENP1-TAP::HIS3MX* | Open biosystems |
| Enp1-TAP  P*_GAL1_-TSR2* | *MATa ura3 leu2 TRP1 ENP1-TAP::HIS3MX Gal1-TSR2::natNT2* | ([Schütz et al., 2014](#_ENREF_8)) |
| Enp1-TAP  P*_GAL1_-FAP7* | *MATa ura3 leu2 TRP1 ENP1-TAP::HIS3MX Gal1-FAP7::natNT2* | This study |
| Noc4-TAP | *MATa ura3 leu2 TRP1 NOC4-TAP::HIS3MX* | Open biosystems |
| Rio2-TAP | *MATa ura3 leu2 TRP1 RIO2-TAP::HIS3MX* | Open biosystems |
| Asc1-TAP | *MATa ura3 leu2 TRP1 ASC1-TAP::HIS3MX* | Open biosystems |
| Fap7-GFP | *MATa his3 leu2 met15 ura3 FAP7-GFP::HIS3MX* | Open biosystems |
| uS11-GFP | *MATa his3 leu2 met15 ura3 RPS14A-GFP::HIS3MX* | Open biosystems |
| *yrb2∆* | *MATa his3 leu2 met15 ura3 YRB2::KANMX* | Open biosystems |
| *prp20-1* | *MATα*, *ura3 leu2 his3 prp20-1::TRP1* | ([Schlenstedt et al., 1997](#_ENREF_7)) |
| *rna1-1* | *MATα*, *ura3 leu2 his3 rna1-1::TRP1* | ([Schlenstedt et al., 1997](#_ENREF_7)) |
| *pse1-1* | *MATa*, *leu2 trp1 URA3::pse1-1 PSE1::HIS3* | ([Seedorf and Silver, 1997](#_ENREF_9)) |
| *kap104∆* | *MATα trp1 ura3 leu2 lys2 KAP104::HIS* | ([Aitchison et al., 1996](#_ENREF_1)) |
| *pse1-1 kap104∆* | *MATa ura3-52 leu2delta1 his3delta200 trp1delta63 pse1-1 HIS3 kap104::TRP1* | ([Schütz et al., 2014](#_ENREF_8)) |
| *kap123∆* | *MATα*, *ura3 leu2 his3 KAP123::TRP1* | ([Schlenstedt et al., 1997](#_ENREF_7)) |
| *msn5∆* | *MATa his3 leu2 met15 ura3 MSN5::KANMX* | Open biosystems |
| *kap114∆ sxm1∆* | *MATα*, *ura3 trp1 KAP114::LEU2 SXM1::HIS3* | ([Fries et al., 2007](#_ENREF_3)) |
| *sxm1∆ kap120∆ nmd5∆* | *MATα*, *ura3 KAP114::LEU2 SXM1::HIS3 NMD5::TRP1* | ([Fries et al., 2007](#_ENREF_3)) |

**Supplementary File 1B - Plasmids used in this study**

| **Plasmid** | **Relevant markers** | **Source** |
| --- | --- | --- |
| pRS425-*FAP7* | *FAP7 2µ LEU2 AMP* | this study |
| pRS426-*RPS14A* | *RPS14A 2µ LEU2 AMP* | this study |
| pRS425-*FAP7-2* | *FAP7-2 D82H84A 2µ LEU2 AMP* | this study |
| pRS316-*RPS2*-*eGFP (uS5)* | *RPS2-eGFP CEN URA3 AMP* | ([Milkereit et al., 2003](#_ENREF_5)) |
| pRS425-*TSR2* | *TSR2 2µ LEU2 AMP* | ([Schütz et al., 2014](#_ENREF_8)) |
| pRS426-*RPS26A* | *RPS26A 2µ LEU2 AMP* | ([Schütz et al., 2014](#_ENREF_8)) |
| pNOPGFP1L-*FAP7* | *GFP-FAP7 LEU2 AMP* | this study |
| pNOPGFP1L-*RPS14A* | *GFP-RPS14A LEU2 AMP* | this study |
| pColA-GST-*FAP7_RPS14A* | *GST-FAP7 RPS14A KAN* | this study |
| pColA-GST-*FAP7_RPS14A-3R* | *GST-FAP7 RPS14 R103R107R114D KAN* | this study |
| pGEX-6P-*RPS14A* | *GST-RPS14A AMP* | this study |
| pColA-*HIS_6_-FAP7* | *HIS_6_-FAP7 KAN* | this study |
| pColA-*HIS_6_-FAP7-2* | *HIS_6_-FAP7 D82H84A KAN* | this study |
| pColA-*HIS_6_-FAP7_RPS14A* | *HIS_6_-FAP7 RPS14A KAN* | this study |
| pColA-*HIS_6_-FAP7_RPS14A-3R* | *HIS_6_-FAP7 RPS14A R103R107R114D KAN* | this study |
| pETduet1-*HIS_6_-TSR2* | *HIS_6_-TSR2 AMP* | ([Schütz et al., 2014](#_ENREF_8)) |
| pETduet1-*RPS26A^FLAG^* | *RPS26A^FLAG^ AMP* | ([Schütz et al., 2014](#_ENREF_8)) |
| pGEX-4TEV-*PSE1* | *GST-PSE1 AMP* | ([Fries et al., 2007](#_ENREF_3)) |
| pGEX-4TEV-*KAP104* | *GST-KAP104 AMP* | ([Maurer et al., 2001](#_ENREF_4)) |
| pGEX-4TEV-*KAP123* | *GST-KAP123 AMP* | ([Fries et al., 2007](#_ENREF_3)) |
| pGEX-4TEV-*KAP95* | *GST-KAP95 AMP* | ([Maurer et al., 2001](#_ENREF_4)) |
| pGEX-4T-*SXM1* | *GST-SXM1 AMP* | ([Caesar et al., 2006](#_ENREF_2)) |
| pGEX-4T-*NMD5* | *GST-NMD5 AMP* | ([Caesar et al., 2006](#_ENREF_2)) |
| pGEX-5G-*KAP120* | *GST-KAP120 AMP* | ([Caesar et al., 2006](#_ENREF_2)) |
| pGEX-4T-*KAP114* | *GST-KAP114 AMP* | ([Caesar et al., 2006](#_ENREF_2)) |
| pGEX-4T-*PDR6* | *GST-PDR6 AMP* | ([Caesar et al., 2006](#_ENREF_2)) |
| pGEX-4TEV-*MTR10* | *GST-MTR10 AMP* | ([Caesar et al., 2006](#_ENREF_2)) |
| pGEX-4T-*MSN5* | *GST-MSN5 AMP* | ([Caesar et al., 2006](#_ENREF_2)) |
| pQE9-*GSP1Q71L* | *HIS_6_-GSP1 AMP* | ([Maurer et al., 2001](#_ENREF_4)) |
